# Does tiotropium lower exacerbation and hospitalization frequency in COPD patients: results of a meta-analysis

**DOI:** 10.1186/1471-2466-10-50

**Published:** 2010-09-21

**Authors:** Ann Van den Bruel, Jeannine Gailly, Mattias Neyt

**Affiliations:** 1 Belgian Health Care Knowledge Centre (KCE), Brussels, Belgium; 2 Academic Centre for Primary Care, KULeuven, Leuven, Belgium; 3 CEBAM, Belgian Centre for Evidence Based Medicine, Leuven, Belgium

## Abstract

**Background:**

International guidelines recommend long-acting bronchodilators in patients who remain symptomatic despite adequate treatment with short-acting bronchodilators. The purpose of this study is to estimate the effect of tiotropium, a long-acting anticholinergic inhalant, on exacerbation and hospitalisation frequency.

**Methods:**

Electronic databases (Medline, Embase, INAHTA, CRD databases, and the Cochrane Library) were searched for randomised controlled trials, comparing tiotropium to placebo, or other bronchodilators. Outcomes were the exacerbation frequency and hospitalisation frequency. Data were pooled using the generic inverse variance method for continuous outcomes.

**Results:**

Nine studies reported comparisons with placebo (n = 8), ipratropium (short-acting anticholinergic inhalant, n = 1), and salmeterol (long-acting β_2_-agonist inhalant, n = 1). Only two studies reported adequate concealment of allocation. Tiotropium reduces the number of exacerbations per patient year by 0.31 (95% CI 0.46- 0.17) compared to placebo, and by 0.23 (95% CI 0.31- 0.15) compared to ipratropium. A significant difference in exacerbation frequency between tiotropium and salmeterol was found (-0.16; 95% CI -0.29 - -0.03) based on approximations of the results of one study.

The number of hospitalisations is reduced by 0.04 (95% CI 0.08- 0.01) per patient year compared to placebo and by 0.06 (95% CI -0.09 - -0.03) per patient year compared to ipratropium.

**Conclusions:**

Statistically significant but clinically small effects were found for tiotropium compared to placebo and ipratropium. The comparison with salmeterol is significant for exacerbation frequency but not for hospitalisation frequency. Publication bias may be present.

## Background

Tiotropium (Spiriva^®^) is a once-daily inhaled long-acting anticholinergic bronchodilator, used for the maintenance treatment of COPD. Next to tiotropium, other long-acting bronchodilators are salmeterol, arformoterol and formoterol, both β_2_-agonists. International guidelines recommend long-acting bronchodilators in patients who remain symptomatic despite adequate treatment with short-acting bronchodilators [1].

Previous meta-analyses on tiotropium have focused on the proportion of patients having at least one exacerbation or COPD-related hospitalisation, and found that tiotropium significantly lowers both outcomes compared to placebo or ipratropium[2,3]. However, results were significantly influenced by the duration of follow-up[4]. This is not surprising, as patients may have several events during follow-up and the likelihood of having at least one exacerbation or COPD-hospitalisation increases with time[5]. Also, treatment is more likely to reduce the number of exacerbation or COPD-related hospitalisations, rather than keeping patients totally exacerbation-free and out of the hospital.

The goal of this study was therefore to estimate the efficacy of tiotropium on exacerbation frequency and COPD-related hospitalisation frequency.

## Methods

### Literature search

The systematic review was iterative: good-quality systematic reviews were searched first; the original studies included in these systematic reviews were complemented with studies published up until November 2008. Databases searched were Medline, Embase, INAHTA, CRD HTA, NICE, the Cochrane Database of Systematic Reviews (CDSR), and CRD DARE. All search terms used are listed in table 1.

**Table 1:** Search terms

	Evidence synthesis	Original studies
**CRD HTA**	tiotropium OR BA 679 BR OR spiriva OR oxitropium'	

**INAHTA**	tiotropium OR BA 679 BR OR spiriva OR oxitropium'	

**NICE**	tiotropium OR BA 679 BR OR spiriva OR oxitropium'	

**CDSR**	'(("tiotropium "[Substance Name]) OR BA 679 BR OR spiriva OR oxitropium) AND systematic[sb]	

**CRD DARE**	'(("tiotropium "[Substance Name]) OR BA 679 BR OR spiriva OR oxitropium) AND systematic[sb]	

**Medline**	'(("tiotropium "[Substance Name]) OR BA 679 BR OR spiriva OR oxitropium) AND systematic[sb]	(("tiotropium "[Substance Name]) OR BA 679 BR OR spiriva OR oxitropium) AND ((clinical[Title/Abstract] AND trial[Title/Abstract]) OR clinical trials[MeSH Terms] OR clinical trial[Publication Type] OR random*[Title/Abstract] OR random allocation[MeSH Terms] OR therapeutic use[MeSH Subheading])

**Embase**		'tiotropium bromide'/exp OR (BA 679 BR) OR 'spiriva'/exp AND [humans]/lim AND [2006-2007]/py

In addition to published studies, attempts were made to identify unpublished studies by searching the FDA http://www.fda.gov/cder/index.html and EMEA websites http://www.emea.europa.eu/htms/human/epar, clinical trial registries, contacting known experts in the field and the manufacturer of tiotropium.

### Selection criteria

Randomised controlled trials with a follow-up of at least 12 weeks after randomisation were eligible if they included a population with stable COPD (no exacerbation one month prior to study entry), and compared tiotropium to placebo, ipratropium bromide or long-acting β_2_-agonists, on exacerbation and COPD-related hospitalisation frequency. Quality of systematic reviews was assessed using the checklist for systematic reviews of the Dutch Cochrane Centre http://www.cochrane.nl. Only reviews with a sensitive search strategy in several databases, and explicit criteria for inclusion and exclusion were eligible. Original studies were assessed for quality using the tool described in the Cochrane Handbook of Systematic Reviews. Original studies were not excluded based on quality assessment. No language restrictions were applied.

Eligibility of studies was assessed by two researchers independently (AVDB, JG). Disagreement was resolved by consensus.

### Analysis

The results of the studies were extracted from the papers by two independent researchers (AVDB, JG). Authors and the drug's manufacturer were contacted in case of missing data.

Outcomes were the number of exacerbations per patient year and the number of hospitalisations per patient year.

Data were pooled using the fixed effects model using the generic inverse-variance approach when no heterogeneity was apparent (I^2 ^≤ 25%)[6]. In all other cases, a random effects model was used.

Funnel plots were constructed when five or more studies were available for one specific comparison and one particular outcome. Publication bias was statistically tested using the Egger's test when ten or more studies were available.

All analyses were performed with Review Manager version 4.2[7].

## Results

### Included studies

The search for systematic reviews identified five studies in the CDSR database, 11 studies in Medline, six in the CRD DARE database and six in the CRD HTA database. Of these, four were potentially relevant based on title and abstract[2,3,8,9]. One study was excluded,[9] because it was not based on a systematic search. In contrast, the quality of the two Barr reviews[2,3,8,9] and the Rodrigo review[2,3,8,9] was very good. However, only one systematic review included trials with a minimum duration of 12 weeks,[8] which was thus included in our study.

The search for original studies was limited to studies published after the literature search of the Barr review (2005). Discarding duplicates, a total of 353 studies were identified in Medline and Embase. After applying inclusion and exclusion criteria on title and abstract, 25 studies were potentially relevant. After assessment in full text, seven studies were included in the final review[10-16]. No report on tiotropium was found on the EMEA site, whereas the FDA published an approval review in 2004 including six clinical trials, corresponding to three published studies, already captured by our literature search.

**Figure 1:**
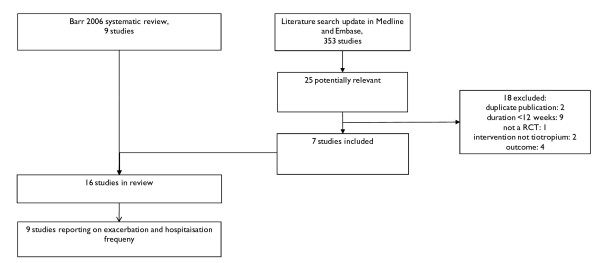
**Flow chart of literature search**.

**Figure 2:**
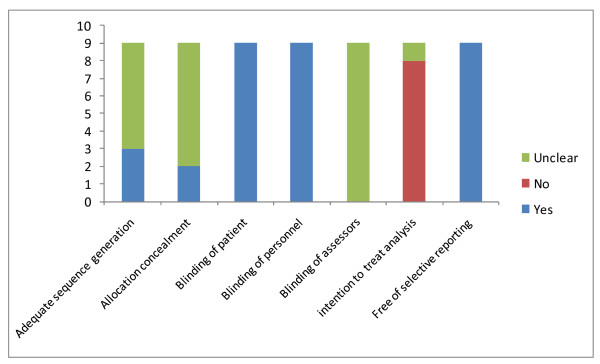
**Quality assessment of included studies**.

In summary, adding the more recent studies to those already included in the review by Barr et al., 16 studies were eligible of which nine reported data that were used in the analyses presented here (see Figure 1 for flow chart of literature search). A description of the characteristics of each study is provided in table 2, and quality assessment is summarised in figure 2.

**Table 2:** Characteristics of included studies

Study	Participants	Interventions and duration	Notes
Brusasco 2003[17]	1207 patients >40 years, >10 pack years, FEV_1 _≤ 65% and FVC ≤ 70%	Tiotropium 18 μg Salmeterol 50 μg Placebo 6 months	Permitted: not stated Not permitted: not stated

Casaburi 2002[21]	921 patients ≥ 40 years, ≥ 10 pack years, FEV_1 _≤ 65%, FVC ≤ 70%	Tiotropium 18 μg Placebo 1 year	Permitted: Albuterol as needed, stable doses of theophylline, inhaled glucocorticosteroids and the equivalent of 10 mg oral prednisone/day, Not permitted: anticholinergics and long-acting β_2_-agonists

Chan 2007[11]	913 patients ≥ 40 years, >10 pack years, FEV_1 _≤ 60% and FEV_1_/FVC ≤ 0.70, ≥ 1 exacerbation in the past 2 years but not within 6 weeks before study	Tiotropium 18 μg Placebo 1 year	Permitted: oral steroids at stable dose ≤ 10 mg prednisone/day, stable doses of inhaled steroids, theophylline, mucolytic, long-acting β_2_-agonists; salbutamol for acute relief Not permitted: inhaled anticholinergics or oral β_2_-agonists

Dusser 2006[22]	1010 patients ≥ 40 years, 10 pack years, pre-bronchodilator FEV_1 _30-65% and FEV1/SVC ≤ 70%, ≥ 1 exacerbation in the last year but not within 6 weeks prior to the study	Tiotropium 18 μg Placebo 48 weeks	Permitted: short-acting betagonists, concomitant use of inhaled or oral steroids (< 10 mg prednisone equivalent) at stable dosages, treatment of COPD exacerbations as deemed necessary Not permitted: Longacting β_2_-agonists, inhaled anticholinergics other than the study drug and theophylline

Niewoehner 2005[18]	1829 patients ≥ 40 years, ≥ 10 pack-years, FEV1 ≤ 60% FVC ≤ 70%	Tiotropium 18 μg Placebo 6 months	Permitted: Usual care authorized (including inhaled corticosteroids and long-acting β-agonists), antibiotics and systemic steroids for exacerbations. Not permitted: other anticholinergic bronchodilators

Powrie 2007[10]	142 patients ≥ 10 pack years, FEV1 < 80% and FEV1/FVC < 70%	Tiotropium 18 μg Placebo 1 year	Permited: usual medication Not permitted: Anticholinergics other than the study drug

Tashkin 2008[13]	5992 patients ≥ 40 years, ≥ 10 pack years, FEV_1 _≤ 70% and FEV_1_/FVC < 0.7, and perform satisfactory spirometry	Tiotropium 18 μg Placebo 4 years	Permitted: usual medication Not permitted: anticholinergics other than study drug unless for the treatment of exacerbations

Tonnel 2008[14]	554 patients ≥ 40 years, > 10 pack years, FEV_1 _≤ 70% and FEV_1_/SVC < 0.7	Tiotropium 18 μg Placebo 9 months	Permitted: salbutamol, stable dosage of theophylline, mucolytics, ICS and oral steroids (< 10 mg of prednisone) Not permitted: β-blockers, antileukotrienes, oral or inhaled long-acting β_2_-agonists, short-acting anticholinergics, or any other investigational drug

Vincken 2002[19]	535 patients ≥ 40 years, ≥ 10 pack-years, FEV1 ≤ 65% and FVC ≤ 70%	Tiotropium 18 μg Ipratropium 40 mg 52 weeks	Permitted: salbutamol as needed; theophyllines, inhaled steroids and oral steroids (at a dose of ≤ 10 mg/day prednisolone or equivalent) if stable dosage. Not permitted: other β_2_-agonists (long or short acting) and inhaled anticholinergic medications (other than study drugs)

Overall, studies were very alike with similar inclusion and exclusion criteria. Patients were at least 40 years old, had smoked at least 10 pack years and suffered from moderate to severe COPD. The patients' mean age ranged from 63.6 to 77.4 years, the baseline FEV_1 _ranged from 36% predicted to 51% predicted.

Duration of the study varied between six months (n = 2), nine months (n = 1), 12 months (n = 5), and 48 months (n = 1). This last study was also the largest study, including 5992 patients[13].

Concomitant medication was specified in all studies except in one[17]. No study allowed the use of other anticholinergic medication. All studies allowed short-acting β_2_-agonist medication, long-acting β_2_-agonists were allowed in four studies, inhalant glucocorticosteroids and oral glucocorticosteroids in all studies, with a maximum dose specified for the latter.

Only two trials[13-15,18] reported adequate concealment of allocation and most studies did not perform an intention to treat analysis for all outcomes reported. Although all studies reported to be double blind, implying blinding of both patient and treating physician, not one study described blinded assessment of the outcome. All studies were sponsored by the pharmaceutical company marketing tiotropium or the comparator drug, and analyses were performed by the pharmaceutical company in two cases.

### Meta-analyses

Studies were identified that compared tiotropium (long-acting anticholinergic) to placebo, ipratropium (short-acting anticholinergic), and salmeterol (long-acting β_2_-agonist).

#### Exacerbation frequency

Most studies defined exacerbations as at least one or two new or increased respiratory symptoms, such as cough, wheeze, dyspnoea, chest congestion, shortness of breath or sputum production, that necessitate a change in treatment. Two studies used a purely symptom-based definition not necessarily leading to a change in treatment[10,19].

For exacerbation frequency, expressed as the number of exacerbations per patient year, results from nine studies were available, of which seven compared tiotropium with placebo and one each with ipratropium and salmeterol.

**Figure 3:**
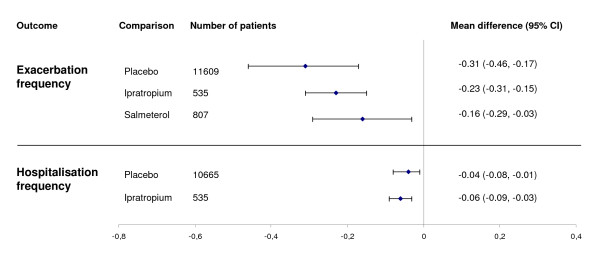
**Summary graph of meta-analyses of continuous outcomes**.

The pooled mean difference between tiotropium and placebo was -0.31 exacerbations per patient year (95% CI -0.46 - -0.17).(Figure 3) However, heterogeneity was substantial (I^2 ^91.2%), mainly caused by one study[10]. This study reported a markedly higher exacerbation frequency in the control group than the other studies (2.46 versus 0.83-1.05 exacerbations per patient year), possibly caused by the purely symptom-based definition of an exacerbation. Heterogeneity decreased slightly after exclusion of this study (I^2 ^= 79%), with a pooled mean difference of -0.19 (95% CI -0.28 - -0.09) exacerbations per patient year.

**Figure 4:**
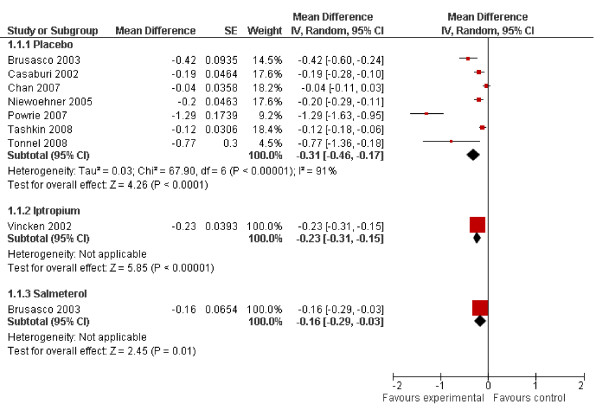
**Meta-analyses on exacerbation frequency**.

Compared to ipratropium, tiotropium reduced the exacerbation frequency with 0.23 (95% CI -0.31 - -0.15) exacerbation per year. Of note, the two studies comparing tiotropium with salmeterol both reported non-significant p-values. However, one study[20] did not detail the exact results, nor were they obtained after contact with the corresponding author and the drug's manufacturer (sponsor of the trial). Consequently, only one study was included in the analysis, resulting in a mean difference of -0.16 exacerbations per patient year (95% CI -0.29 - -0.03).(Figure 3) Although the paper reported a non-significant p-value, we obtained a significant 95% CI, most likely caused by differences in rounding in the derivation of the standard error from the p-value. Detailed results are presented in Figure 4.

#### COPD-related hospitalisation frequency

Six studies reported sufficient information on hospitalisation frequency to be included in the meta-analysis, five comparing with placebo[11,13,18,21,22] and one with ipratropium[19].

**Figure 5:**
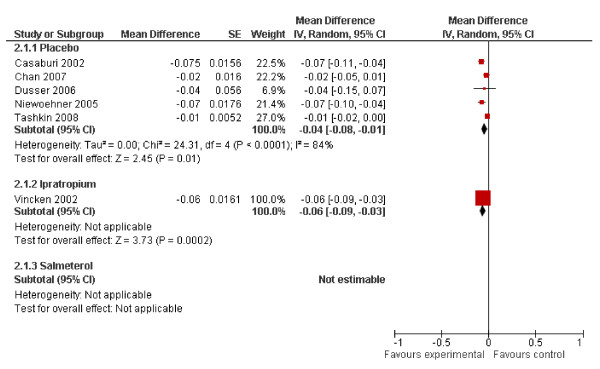
**Meta-analyses on COPD-related hospitalisation frequency**.

The difference in hospitalisation frequency with placebo was -0.04 per patient year (95% CI -0.08 - -0.01), and with ipratropium -0.06 (95% CI -0.09 - -0.03).(Figure 3) The frequency in the control group ranged from 0.150-0.250 per patient year. Two studies comparing tiotropium with salmeterol reported non-significant p-values, but no exact results. Again, these could not be obtained after contacting authors and manufacturer. (Detailed results Figure 5)

#### Publication bias

**Figure 6:**
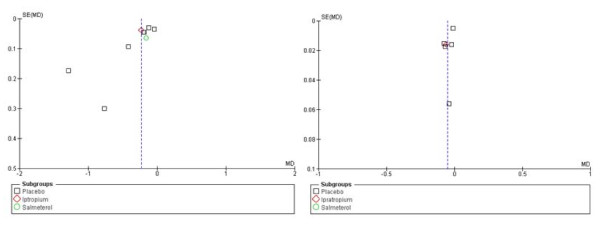
**Funnel plots of studies reporting exacerbation frequency (left) and exacerbation-related hospitalisation frequency (right)**.

Funnel plots were constructed, but formal testing was not possible because less than 10 studies were available for either comparison (Figure 6). The funnel plot on exacerbations showed asymmetry, suggesting a lack of studies reporting less favourable results.

## Discussion

Tiotropium lowers the number of exacerbations per patient year significantly by 0.31 (95% CI -0.46 - -0.17) exacerbations/year compared to placebo, and by 0.23 (95% CI -0.31 - -0.15) exacerbations/year compared to iptratropium.

Compared to salmeterol, we found a statistically significant difference of 0.16 (95% CI -0.29 - -0.03) exacerbations/year based on one single study. However, this comparison was reported as non-significant by the original authors and may be significant in our analyses because of the approximations we had to use. In addition, a second study reporting a non-significant difference but not detailing the results could not be included in the analyses. For these reasons, this result should be treated with great caution.

In addition, there was a statistically significant effect on the number of hospitalisations per patient year of 0.04 (95% CI -0.08 - -0.01) compared to placebo, and 0.06 (95% CI -0.09 - -0.03) compared to ipratropium.

The robustness of these findings is influenced by the relative moderate quality of the individual studies - only two studies reported adequate concealment of allocation- and evidence for publication bias was found.

A recent meta-analysis by Kesten et al. (2009)[23] comparing tiotropium to placebo found a smaller difference of -8.90 (95% CI -11.0- (-6.83)) per 100 patient years. This study is not based on a systematic review of the literature, but used all phase III and IV studies in the Boehringer database.

In a recent network meta-analysis, combining both direct and indirect evidence on different bronchodilators, long-acting anticholinergics, long-acting β_2_-agonists and the combination of long-acting β_2_-agonists and inhaled corticosteroids were found to significantly reduce the number of patients with at least one exacerbation but without significant differences between them,[24] which is consistent with our findings.

Our study has some limitations. Publication bias may be present, by which studies with less favourable results are not published[25] and consequently not included in the analyses. Our meta-analysis was further limited by the fact that not all results were available for inclusion, although attempts were made to obtain all data. Consequently, the analyses for salmeterol are incomplete. In addition, the quality of the original studies was not optimal. Especially the uncertainty on allocation concealment increases the risk of bias. Finally, we were not able to analyse results according to COPD severity. It might be possible that some patients would benefit more from treatment with tiotropium than the general COPD population. An individual patient data analysis might be able to explore the influence of patient characteristics on efficacy.

## Conclusion

Patients taking tiotropium experience 0.3 exacerbations less per year compared to placebo and 0.2 compared to iptratropium. Compared to salmeterol, tiotropium users experience 0.16 exacerbations less per year, although this result should be treated with caution due to incomplete results and approximations.

In addition, the number of hospitalisations per patient year is reduced by 0.04 compared to placebo. No effect was found compared with salmeterol. The results may be influenced by flaws in design and publication bias.

## Competing interests

AVDB, JEG and MAN all state that they have no conflicts of interest that could inappropriately impact their work.

## Authors' contributions

AVDB designed the protocol, performed the searches, selection, data extraction and analyses, and drafted the manuscript. JEG performed the selection and data extraction, and revised the manuscript. MAN assisted in designing the protocol and revised the manuscript. All authors read and approved the final manuscript.

## Note

The corresponding author (AVDB) confirms that she had full access to all the data in the study and had final responsibility for the decision to submit for publication.

## Pre-publication history

The pre-publication history for this paper can be accessed here:

http://www.biomedcentral.com/1471-2466/10/50/prepub
